# In the Elderly, Failure to Update Internal Models Leads to Over-Optimistic Predictions about Upcoming Actions

**DOI:** 10.1371/journal.pone.0051218

**Published:** 2013-01-09

**Authors:** Gilles Lafargue, Myriam Noël, Marion Luyat

**Affiliations:** 1 Département de Psychologie, Université Lille Nord de France (Lille 3), Villeneuve d'Ascq, France; 2 Laboratoire de Neurosciences Fonctionnelles et Pathologies, Université Lille Nord de France, Lille, France; 3 CHR de Roubaix, Hôpital Victor Provo, Pavillon de Médecine Gériatrique, Roubaix, France; McGill University/Douglas Mental Health Univ. Institute, Canada

## Abstract

Before an action is performed, the brain simulates the body's dynamic behavior in relation to the environment, estimates the possible outcomes and assesses the feasibility of potential actions. Here, we tested a hypothesis whereby age-related changes in sensorimotor abilities result in failure to update internal models of action in the elderly. Young and older adults were required to judge in advance whether or not they could stand on an inclined plane (Experiment 1). Relative to young adults, elderly adults overestimated their postural capabilities: although the two groups made similar feasibility judgments, elderly participants showed significantly worse postural performance levels. This tendency to overestimate their own ability persisted when elderly adults had to not only estimate the feasibility of an action but also endanger themselves by walking towards an obstacle that was too high for them to clear (Experiment 2). An age-related failure to update internal models may prompt the elderly to make over-optimistic predictions about upcoming actions. In turn, this may favor risky motor decision-making and promote falls.

## Introduction

Each year, one in three people over the age of 65 will suffer a fall. In 5% to 15% of cases, the fall will result in severe trauma or even death [1], [2]. Several risk factors for falls have been identified; they include diseases (e.g. neurological, cardiovascular, gastrointestinal and metabolic diseases), sensory impairments (particularly visual impairments), muscle- and joint-related problems, the lack of attentiveness associated with anxiety or depression and the effects of certain medications [1], [3]. The biomechanical causes of loss of balance have also been extensively investigated [4], [5]. However, the sum of these factors fails to fully explain the increasing occurrence of falls among the elderly.

In the field of cognitive neuroscience, it is generally accepted that before an action is performed, the brain simulates the body's dynamic behavior in relation to the environment and estimates the possible outcomes and consequences [6], [7]. The ability to predict the effects of an action is a fundamental brain function and is critical in optimizing motor decisions [7] and judging the feasibility of potential actions [8], [9].

Under normal circumstances, the brain continuously updates its internal models of action on the basis of the person's experience [7], [10]. If this updating does not occur properly, there will be a discrepancy between what the person believes she/he can do and what she/he still can really do. In older adults, internal models must take account of the age-related declines in sensorimotor [11] and physical abilities [12], [13], [14], [15], [16]. Failure to update internal models might result in over-optimistic predictions about upcoming behaviors. Thus, in certain cases, older adults may not be able to correctly evaluate their motor and postural capabilities. For example, they may attempt to walk on surfaces that appear to be danger-free but on which they would be unable to stand (in view of impaired motor skills). Hence, the overestimation of postural capabilities may be a major risk factor for falls in the elderly.

Motor imagery can be considered as the off-line recruitment of neural networks involved in perception and action [6], [17], [18]. Studies of regional cerebral blood flow have shown that most of the brain areas activated during overt movement (such as the parietal, motor and premotor cortices, the basal ganglia and the cerebellum) are also activated during motor imagery [19], [20], [21], [22], [23], [24]. One way of investigating the unconscious process of action representation and internal models of action is conscious motor imagery, which consists in imagining oneself in action while inhibiting the motor output [6].

The main purpose of the present study was to determine (through the use of two tasks with real-world relevance) whether overestimation of one's postural capabilities is characteristic of elderly adults and, if so, whether this overestimation could prompt them to adopt riskier locomotor behavior. As mentioned above, we reasoned that the elderly's decreased postural and physical abilities create a set of conditions in which internal models of action are not correctly updated. This view is supported by experimental evidence of age-related changes in the elderly: a loss of accuracy in predictions of hand movements through motor imagery [25], [26], [27], [28], [29], a decreased ability to imagine movements from an internal perspective [30] and a tendency to overestimate the boundaries of prehensile space [17]. Interestingly, it has also been found that patients with Parkinson's disease [31] and hemiplegic patients [32] overestimate their reaching abilities (relative to healthy controls).

In a first experiment with young and elderly adults, we compared verbal predictions about the feasibility of standing on an inclined plane surface with postural performance levels (i.e. the steepest incline on which each participant could actually stand upright). In a second experiment, we compared verbal predictions with the actual ability to step over a bar set at different heights. In this latter experiment, the participants not only had to give verbal responses but were also required to immediately commit themselves to a real action by attempting to step over the highest bar they believed they could clear. Our aim was to directly test the hypothesis whereby elderly adults are more likely than younger adults to endanger themselves (by walking towards an obstacle that is too high for them to clear, for instance).

## Experiments

The study protocol was approved by the local investigational review board (CPP Nord-Ouest IV, EudraCT identifier: 2007-A01148-45). All participants gave their prior, written informed consent and all data were recorded anonymously.

The participants in the “young adult” group were University of Lille undergraduates who had replied to advertisements placed around campus. In the “older adult” group, half were spouses of patients attending the Geriatric Medicine Unit at Roubaix Hospital and half were recruited through advertisements in local senior citizens' groups. All participants had normal vision or corrected-to-normal vision with contact lenses or glasses. However, normal visual acuity was checked immediately before the experiment by using a Snellen eye chart at 3 meters. None of the participants had a history of neurological, vestibular or motor disorders. To detect any cognitive deterioration, each elderly participant underwent a neuropsychological assessment (including the Mini Mental State Examination (MMSE)) [33]. Participants with an MMSE score below 28 were excluded from the study.

### Experiment 1

#### Participants

Twenty young adults (8 women and 12 men; mean ± SD age: 24.40±4.17; range: 18–32) and twenty elderly adults (12 women and 8 men; mean ± SD age: 73.45±6.10; range: 66–84) took part in the experiment.

### Materials and procedure

#### The main experiment: self-estimated ability

On the basis of a mental simulation (i.e. without actually performing the action), each participant had to judge (and then state verbally) whether he/she would be able to stand on an inclined plane (95 cm×90 cm) for at least 5 seconds without bending his/her knees or bending at the waist. Given the age-related decrease in the efficiency of sensory functions in general and vision in particular and in order to increase our data's reliability, we tested visual and haptic exploration modes. In the latter mode, the participant was blindfolded and explored the incline plane with a cane. For each presented slope, the participant gave her/his response (yes/no) and stated her/his level of confidence in that judgment on a scale ranging from 0 (low) to 7 (high). Each slope (ranging from 6° to 48° in inclination, with a 6° increment) was presented four times, in random order. The response time (the time interval between presentation of the slope and the participant's reply) was also noted.

#### The control experiment: actual ability

We next measured actual performance by filming the participant on the inclined plane. The results were analyzed off-line for loss of balance by two independent observers and coded as (1) bending the knees or bending at the waist, (2) refusal to step onto the plane (3) sliding off, (4) stepping off the plane and (5) maintenance of an upright stance for at least 5 seconds. The planes were presented in order of increasing (“uphill”) inclination, with the slope parallel to the body midline. Each slope was presented twice. No external assistance was given but an investigator was positioned so as to catch the participant in the event of a slip or a fall. The test ended when the participant failed to stay on the inclined plane for at least 5 seconds or refused to step onto the inclined plane (with two trials for each inclination). Postural ability was measured once with the participant blindfolded (the “no vision” condition) and once not blindfolded (the “vision” condition). Interrater reliability was near-perfect (r = 0.97). For the few cases in which the observers disagreed on the postural threshold (see the next section), we took the mean of the observers' respective values.

### Results

For each subject, we determined a “feasibility threshold” as a self-estimation of ability. This threshold corresponded to the critical slope for which we obtained a positive response (“yes, I can stand upright on that slope”) in 50 percent of the cases (each inclination was presented four times). It was obtained using the following equation:
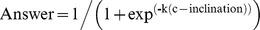
where *c* is the critical slope (in °) with a 50% “yes” response rate (Answer = 50%), *k* is the slope of the curve at the point where Answer = 50% and *inclination* is the inclination of the plane. For each series, a “postural threshold” (for actual ability) was defined as the steepest slope on which the participant could stand for at least 5 seconds while complying with the four above-mentioned criteria. The mean postural threshold was computed by averaging the results for the two trials with each slope. We also computed an “overestimation index” by subtracting each participant's mean postural threshold from his/her mean feasibility threshold.

**Figure 1:**
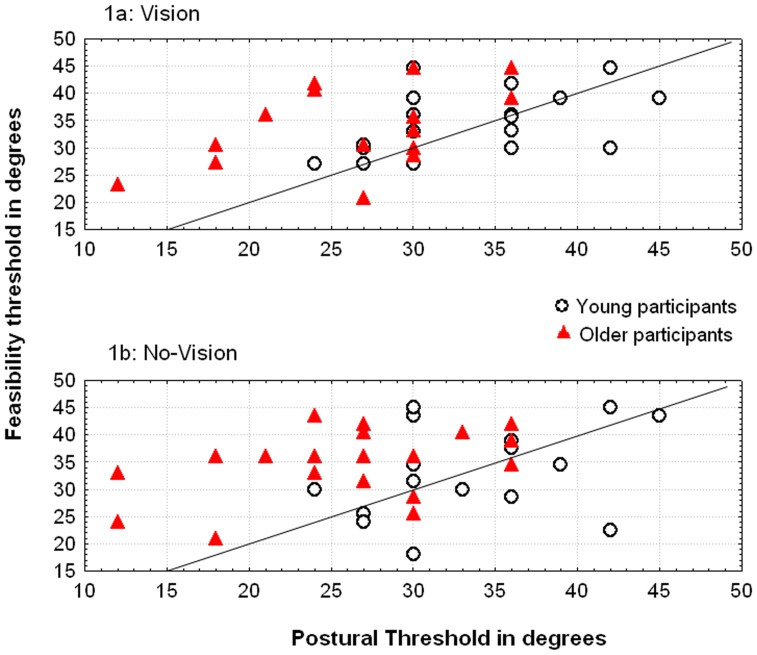
Individual results. Self-estimated ability (y-axis; feasibility thresholds in degrees) and actual ability (x-axis; postural thresholds in degrees) as a function of age and the vision-enabled and haptic conditions. The diagonal line represents a perfect fit between self-estimated and actual ability; values below the diagonal correspond to underestimation of ability and values above the diagonal correspond to overestimation. NB: although the scatter plots of the young adult and older groups show similar values on the y-axis (self-estimated ability), the plot for the older group is shifted to the left on the x-axis (*i.e.* lower actual ability).

The mean data for each group are presented in Table 1. The individual thresholds derived from mental simulation and postural tasks are depicted in Figure 1.

**Table 1:** The mean (standard deviation) feasibility and postural thresholds in degrees (i.e. self-estimated ability and real ability, respectively), as a function of condition and age.

	Self-estimated ability		Real ability	
	vision	haptic	vision	no vision
**Young adults**	35.50° (6.11)	33.75° (8.63)	33.45° (5.78)	32.40° (6.05)
**Older adults**	34.73° (6.99)	34.73° (6.13)	28.05° (6.83)	25.80° (7.11)

#### Self-estimated ability

We first analyzed self-estimated ability (the y-axis in Figures 1a and b) with a 2×2 analysis of variance (ANOVA: *age* [young, elderly]×*exploration condition* [visual, haptic]) on the feasibility thresholds, with repeated measures on the *exploration condition* and *age* as a category-specific predictor. This analysis did not reveal any significant effects of either *age* (*F*(1, 38) = 0.002; p = .96) or *exploration condition* (*F*(1, 38) = 0.80; p = .38). Furthermore, there was no interaction between the two factors (*F*(1, 38) = 0.80; p = .38). As shown in Figure 1, the elderly adult group (*M* = 34.73°) and young adult (*M* = 34.63°) group did not differ significantly in terms of their self-estimated ability (y-axis) in either visual or haptic exploration modes.

In order to test for possible differences in the participants' ability to reliably detect a slope's “standability”, we analyzed the “threshold” slopes (parameter *c* in the individual psychometric function) with a 2×2 ANOVA (*age* [young, elderly]×*exploration condition* [visual, haptic]), with repeated measures on the *exploration condition* and *age* as a category-specific predictor. Again, neither an effect of *age* (*F*(1,38) = 0.25; p = .62) nor an *age*×*exploration condition* interaction was observed.

#### Actual ability (postural task)

Actual ability (*i.e.* the mean postural threshold: the x-axis in Figures 1a and b) was analyzed in a 2×2 ANOVA (*age* [young, elderly]×*condition* [vision, no vision]), with repeated measures on *condition* and *age* as a category-specific predictor. The analysis revealed a significant effect of *age*: *F*(1, 38) = 9.242, p = .004, η*_p_^2^* = 0.18. Unsurprisingly, the young adult participants were able to stand on steeper slopes (M = 32.93°) than the elderly participants were (M = 26.93°). The ANOVA also revealed an effect of *condition* (*F*(1,38) = 9.51; p = .004, η*_p_^2^* = 0.13). The postural thresholds were higher in the vision condition (*M* = 30.75°) than in the no vision condition (*M* = 29.10°). The interaction between the *age* and *condition* was not significant (*F*(1, 38) = 1.259; p = .27).

#### Accuracy of self-estimation, relative to postural performance

**Figure 2:**
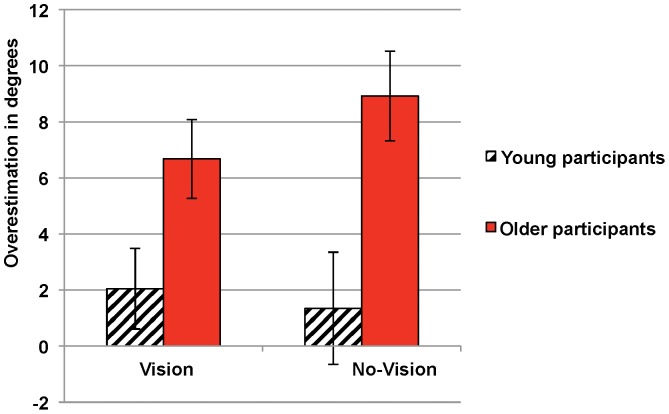
Mean overestimation in each of the groups. The overestimation index was computed by subtracting each participant's mean real postural threshold from his/her mean feasibility threshold. The bars represent standard error of the means.

A 2×2 ANOVA (*age* [young, elderly]×*condition* [vision, no-vision]) on the overestimation index (with repeated measures on *condition* and *age* as a category-specific predictor) confirmed the effect of *age* (*F*(1,38) = 9.29; p = .004, η*_p_^2^* = 0.15). Elderly participants overestimated to a significantly greater extent (*M* = 7.80°), when compared with the young adults (*M* = 1.70°) (see Figure 2). There was neither a significant effect of *condition* nor a significant interaction between *age* and *condition*. Moreover, the proportion of participants overestimating their performance was greater in the group of elderly adults (χ^2^_1_ = 4.29, p = .038 *vs.* the young adults); in the vision condition, 17 of the 20 elderly participants overestimated their performance, whereas only 11 of the 20 young adult participants did so.

### Response times and level of confidence

Analysis of the elderly participants' response times (obtained during the mental imagery task) showed that the mean highest response time (in both exploration modes) was found for a slope of 36° (*F*(1,38) = 19.64; p<.0001); this slope was steeper than the mean postural threshold (*M* = 26.92°) but close to the mean feasibility threshold (*M* = 34.77°).

Analysis of the elderly participants' level of confidence showed that the lowest value was also observed for a slope of 36° (*F*(1,38) = 21.44; p<.0001).

## Discussion

In the motor imagery task, we did not observe a significant effect of age or exploration condition (*i.e.* visual vs. haptic exploration). In contrast to self-estimated ability, we observed a statistically significant difference in real performance (*i.e.* the postural threshold) between young and elderly adult participants. As expected (given the decrease in postural performance with age), elderly participants were less able to cope with steep slopes (under both vision and no-vision conditions) than young adults were. In summary, elderly participants appeared to behave as if they were younger and thus judged that they could stand on a slope that was too steep for them in reality. Overestimation bias was observed under both “vision” and “no vision” conditions (See Figure 2). Although both elderly and younger subjects overestimated their ability (by 7.8° and 1.7° on average, respectively) the overestimation was 4.5 times greater in the elderly adult group. The lower level of postural ability in the “no vision” condition (in the postural task) agrees with the literature data on postural stance [34].

Nevertheless, the existence of a significant relationship between performance overestimation and an increase in falls remains to be established. Indeed, in our experiment, the subjective judgments and the real performance levels were recorded during separate sessions. The participant's subjective judgments about the slope's “standability” were verbal and not linked to an obligation to actually execute the action immediately thereafter. Even though the non-verbal measures (such as the response time) were in line with an overestimation bias, the question is whether or not people who tend to overestimate their performance levels are more likely to take greater locomotor risks. To investigate this hypothesis, we performed a second experiment in which new groups of young and elderly adult subjects were required to commit to an action. They had to walk towards and step over a raised horizontal bar (i.e. a hurdle) but only when they felt able to do so. However, to avoid injury or falls, the investigator feigned a technical problem and stopped the participant just before he/she stepped over the obstacle. Moreover, the overestimation bias seen in Experiment 1 could have been produced by the subjects' lack of day-to-day familiarity with the task (*i.e.* standing on an inclined plane). Thus, the second objective of Experiment 2 was to see whether or not overestimation bias occurred in a task that is more commonly encountered in activities of daily living: stepping over an obstacle.

### Experiment 2

#### Participants

Twenty young adults (12 women and 8 men; mean ± SD age: 23.8±2.7; range: 19–28) and twenty elderly adults (14 women and 6 men: mean ± SD age: 76.1±6.4; range: 66–89) took part in the experiment. The procedure comprised two successively presented tasks.

### Materials and procedure

#### The main experiment: self-estimated ability with potential personal endangerment

Each participant (tested individually) stood upright at a distance of 4 m from the obstacle, which consisted of a horizontal bar that could be supported by (but not attached to) two vertical poles at different heights from the ground. The participant was asked to judge whether they would be able to walk towards and step over the hurdle. When the hurdle was set at the greatest height that the participant felt capable of clearing successfully (i.e. without falling and without knocking the bar off), he/she was told to really perform the action. This task was performed twice. In a decreasing trial, the bar was initially set at a height of 1.60 m and then lowered in 5 cm increments. In an increasing trial, the bar was initially set at a height of 13 cm and then raised successively to heights of 20 cm, 30 cm, 40 cm. Beyond 40 cm, the height increment was 5 cm.

For decreasing trials, the participant was required to mentally simulate the step-over movement and state its feasibility. The height adjustments were stopped when the participant judged that she/he was able to step over the bar. At this moment only, she/he was asked to walk towards the obstacle and really perform the action. Once the participant had approached the hurdle but before he/she had raised his/her leading leg, the experiment feigned a technical problem and stopped the participant from continuing. For increasing trials, the participants were also told to mentally simulate the step-over movement and state its feasibility. Again, the height adjustments were stopped when the participant judged that she/he was not able to step over the hurdle successfully. At this moment only, the hurdle was lowered to the previous height and the participant was asked to walk towards the obstacle and really perform the action. Again, the experiment feigned a technical problem and stopped the participant before he/she had raised his/her leg for the step-over movement.

It should be noted that we could not perform more than two trials of this type (one at the end of the increasing sequence and one at the end of the decreasing sequence) because otherwise the participants became aware of the “trickery”. The greatest height that the participant had attempted to clear was taken as the mean of the “decreasing” value and the “increasing” value.

#### The control experiment: actual ability

The participant was invited to stand upright at a distance of 4 m from the hurdle. She/he was informed that she/he was going to really step over the hurdle. An experimenter stood nearby the hurdle for safety reasons. The bar was first set at a height of 13 cm from the ground. The bar was then placed successively at 20 cm, 30 cm and 40 cm. Beyond 40 cm, the height increment was 5 cm. At each height, the participant was invited to walk towards and then step over the bar without falling and without knocking the bar onto the ground. The participant was free to choose the lead foot for the step-over movement but was told that jumping was prohibited.

If two consecutive trials at a given height were successful (i.e. without falling, without knocking the bar off and without refusing to step over), the bar was moved up a notch. The participant was free to refuse to attempt the step-over if he/she judged that the hurdle was too high. The task was stopped either at the request of the participant (i.e. two refusals to step over the hurdle at the same height) or when the bar was knocked off its supports twice at the same height. We noted the highest hurdle that the participant could clear (i.e. actual ability) in at least one of the two trials at that height.

### Results

#### The potential for risky locomotor behavior

**Figure 3:**
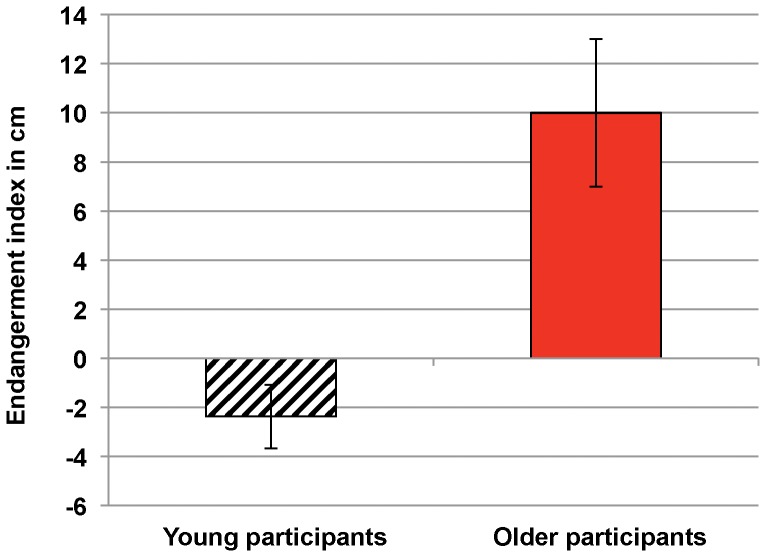
Mean endangerment indexes for both groups. The endangerment indexes were computed by subtracting the greatest height that the participant could really step over from the greatest height that the participant had attempted to clear. The bars represent standard error of the means.

To test whether elderly participants were more inclined to endanger themselves than younger ones, we computed an endangerment index by subtracting the greatest height that the participant could really step over (in the second task) from the greatest height that the participant had judged feasible (*i.e.* measured in the first task, with endangerment). Thus, endangerment was defined as the participant's decision (in the first task) to try to clear over a height which he/she was unable to clear (in the second task). The results (see Figure 3) showed that elderly participants were significantly more likely to choose a hurdle height that was too high for them (*M* = +10 cm; *SD* = 13.45) than the young adult participants (*M* = −2.38 cm, *SD* = 5.82) (t_38_ = −3.78; p = .00005).

### Discussion

The result of our second behavioral experiment confirmed that the elderly adults overestimated their postural performance. Consequently, this overestimation was not linked to the specific features of (or unfamiliarity with) our first task (*i.e.* postural ability on an inclined plane) because it was also observed when stepping over an obstacle - a more common event in daily life. Moreover, the results of this second experiment showed that elderly participants were more likely to commit to potential risky locomotor behavior. Had we not stopped the elderly participants in the second experiment, they would probably have knocked the bar off or even (in view of the high mean degree of overestimation, at *M* = 10 cm) fallen over. It is important to note that on average, the younger participants underestimated their greatest passable height (i.e. there were able to clear a hurdle set above their self-estimated maximum height). This is in line with studies that have reported that healthy young adults tended to underestimate their reaching abilities [35], [36], [37], [38]. Robinovitch hypothesized that this underestimation was a potential safety factor and reduced the risk of losing one's balance [38]. As mentioned above, overestimation in elderly persons suggests a tendency to lose this potential safety factor.

## General Discussion

Our results showed that healthy elderly adults overestimated their physical capabilities in two different experimental tasks. When the elderly participants had to judge whether they could stand on an inclined plane (Experiment 1) or step over an obstacle (Experiment 2) they significantly overestimated their capabilities in most instances. The present study replicated the results of two pilot experiments in which our research group had already observed the elderly's tendency to overestimate capabilities in two variants of the tasks used here [39], [40]. Importantly, the present study involved different participants, a large sample size and additional measures (such as the response time, level of confidence and endangerment index). This demonstrates the robustness of the effect in two tasks with real-world relevance. Moreover, the results obtained in Experiment 2 showed that this overestimation can induce a risk of falls. The elderly participants not only verbally overestimated their capabilities but also endangered themselves by walking towards an obstacle that was too high for them to clear.

As mentioned in the Introduction, it is well known that physical activities and abilities decline with age. In elderly adults difficulty in adjusting to age-related changes in postural affordances might thus result from a decrease in physical exercise and inadequate updating of the diminished motor capabilities. Experiments focusing on the frequency and intensity of involvement in sporting activities in elderly adults would be useful for testing this hypothesis.

On the neurological level, inadequate updating of diminished physical ability in the elderly may be linked to age-related structural changes in the brain, such as the loss of gray and white matter (especially in the frontal and parietal lobes [41]), shrinkage of the basal ganglia [42] and cerebellar atrophy [43]. These brain structures form corticosubcortical systems that are known to be crucial for the acquisition, execution and adaptation of motor skills [44]. Moreover, a decrease in muscle mass and strength during aging modifies the relationships between motor command and limb motion. According to Shadmehr et al. [45], maintenance of a desired level of performance means that the brain has to adapt to these changes by updating internal models of action that predict the sensory consequences of motor commands. In this sense, inadequate updating of the physical capabilities with aging could be viewed as the individual's inability to make use of sensory prediction errors to compute the motor command that initiates subsequent movement. Indeed, damage to the cerebellum [46] and basal ganglia [47] has been shown to impair the use of sensory feedback for adapting movement.

When considered together with the literature data, our results suggest that the outcomes of elderly people's mental simulations do not accurately reflect their diminished physical capabilities. In Experiments 1 and 2, the participants had to mentally simulate the given action before giving their reply (“I can/cannot stand upright on the slope” and “I can/cannot step over the obstacle”, respectively). The fact that the elderly participants behave in much the same way as young adult participants in this respect suggests that motor imagery capacity *per se* (which is underpinned by frontoparietal networks [48]) is relatively unaffected by age. In contrast, we can reasonably suppose that the updating process (a function mediated by the cerebellum and the basal ganglia, as mentioned above) is less efficient in the elderly. A combination of impaired updating and decreased physical ability may lead to over-optimistic predictions about the feasibility of actions.

However, it could also be argued that the elderly participants' overestimation resulted from knowingly being part of an experiment (the Hawthorne effect) or from an intention to impress the investigator. Even if we cannot completely discount this interpretation, a number of observations argue against it. For instance, the participants knew from the outset that Experiment 1 was to be followed by measurement of their true abilities. In other words, the task was not a mere discussion with an investigator but consisted in accurately predicting the participant's subsequent postural capabilities. Interestingly, further analysis of the elderly participants' response times in the perceptual task in Experiment 1 showed that the longest mean values (in both exploration modes) were recorded for a slope of 36°; this slope was steeper than the mean postural threshold (26.92°) but was close to the feasibility threshold (34.77°). The lowest mean level of confidence was also observed for a slope of 36°. Analysis of the psychometric functions did not reveal any significant inter-group differences in terms of discriminative ability. Moreover, the overestimation bias in the elderly persisted in Experiment 2, in which the participant had to freely commit to the action of stepping over the obstacle. Taken as a whole, these non-verbal data suggest that the observed overestimation bias could not be fully ascribed to the participant's intention to impress the investigator.

Another study limitation relates to the high inter-individual variability seen in both young and elderly participants; further research on this topic is needed. Several factors (such as the participant's degree of autonomy in activities of daily living or involvement in sports or exercise) may have a role in this variability. It would be of value to identify the elderly adults who are most inclined to overestimate their postural capabilities.

Our findings may open up perspectives for reducing risky locomotor behavior and thus preventing falls. Fall prevention training could be based on exercises in which the elderly explicitly learn to acknowledge their physical limitations. At present, the detection of people at risk of falls takes account of several intrinsic factors (such as age, health status and the presence of diseases and sensory impairments) and extrinsic factors (such as alcohol consumption, sedentariness or an inappropriate environment) [49]. However, our findings highlight another aspect of this problem: bias in self-perception of one's physical abilities. Further experiments are needed to explore and extend this perspective by establishing whether subjective age, depression or certain personality traits are linked to the emergence of overestimation bias.
